# Evaluating the Citation Accuracy of ChatGPT-5 Using the American Academy of Orthopaedic Surgeons Clinical Practice Guidelines

**DOI:** 10.7759/cureus.113654

**Published:** 2026-07-30

**Authors:** Ethan M Bernstein, Brandon Bol, Jean Shanaa, Anya Ramsamooj, Tony Baldini, Zachary C Lum

**Affiliations:** 1 College of Medicine, California Northstate University, Elk Grove, USA; 2 Orthopedic Surgery, UC Davis School of Medicine, Sacramento, USA

**Keywords:** artificial intelligence, citation accuracy, citations, clinical practice guidelines, large language models

## Abstract

Background: Large language models (LLMs) have demonstrated strong performance in generating medical responses; however, concerns persist regarding the accuracy of citations for these responses. Prior work has shown that earlier models frequently fabricate or misattribute references when queried on American Academy of Orthopaedic Surgeons (AAOS) Clinical Practice Guidelines (CPGs). With rapid advancements in model development, newer-generation LLMs may demonstrate improved citation accuracy and reliability. This study evaluates the citation accuracy of ChatGPT-5 in response to AAOS CPG-based queries.

Methods: A computer-based observational study evaluating the citation accuracy of ChatGPT-5 (December 2025) was conducted from February to March 2026. Seventy recommendations from four AAOS CPGs were converted into standardized prompts, and ChatGPT was asked to generate a list of references to support its claims in response to these prompts. Five independent graders evaluated responses for citation accuracy. Citation elements assessed included title, authorship, journal, year, volume, pages, and PubMed identifier (PMID). Hallucinated references were defined as nonexistent or non-indexed studies.

Results: A total of 350 queries generated 2,736 references, of which 7.13% were fabricated. Citation inaccuracies were most frequent for PMID (37.28%), title (30.70%), and pages (27.49%). Overall, 49.34% of citations were bibliographically accurate, and complete accuracy within a query occurred in 8.00% of cases.

Conclusions: Citation errors and fabricated references persist in ChatGPT-5. Independent verification of generated references therefore remains necessary.

## Introduction

Since its release in 2022, many different uses for large language models (LLMs), such as Chat Generative Pretrained Transformer (ChatGPT, OpenAI), have been proposed. Artificial intelligence (AI) is of interest to almost every field, with different implementations of AI having been published in academic literature among disciplines, such as tourism, education, and medicine [1-3]. ChatGPT has proven competency in proofreading essays, assisting with generating code for computer programmers, and answering medical board-style questions with accuracy [4-8]. Multiple studies have evaluated ChatGPT’s responses to common patient questions and compared these to physicians’ responses; one study reported ChatGPT’s responses to be rated similarly to physicians’ responses, while another reported that participants felt ChatGPT responded with more empathetic and higher quality responses than physicians [9,10]. Similar findings have been reported across multiple linguistic domains, with a recent study finding ChatGPT-4.5 to achieve higher response quality scores compared to community pharmacists in both English and Arabic [11].

Despite ChatGPT’s documented ability to correctly answer medical questions and provide appropriate responses to patient questions, it often struggles to provide accurate and relevant references for its responses, often “hallucinating” by generating completely [12-14]. Regarding Orthopaedic Surgery specifically, ChatGPT’s responses are not consistent and have limited accuracy when compared to the American Academy of Orthopaedic Surgeons Clinical Practice Guidelines (AAOS CPGs) for hip fractures in older adults, rotator cuff injuries, and developmental dysplasia of the hip [15-17]. A recent study published by McCavitt et al. evaluated the accuracy and quality of evidence cited in responses by ChatGPT-4o mini to questions regarding the AAOS CPG for Management of Hip Fracture in Older Adults [18]. Since this study, ChatGPT-5 was introduced as OpenAI’s most advanced system to date, with the development of significant improvements in coding, mathematical calculations, writing assistance, health information, and visual perception [19]. Specifically, ChatGPT-5 is supposed to be more useful for real-world queries compared to previous models. Additionally, it is supposed to be the best model for health-related queries by providing more accurate and precise responses, identifying potential concerns, and acting as an interactive thought partner by asking follow-up questions [19]. 

The primary aim of this study was to evaluate the bibliographic accuracy of citations generated by ChatGPT-5 in response to orthopaedic medical questions. Primary outcomes included the accuracy of individual citation components, the proportion of fully accurate citations, and the rate of fabricated references. Secondary objectives were to determine the proportion of responses in which all generated citations were fully accurate and to contextualize the findings using previously published data from older model versions as historical context.

## Materials and methods

Study design

The investigation was conducted from February 2026 to March 2026 using ChatGPT (GPT-5, paid version, OpenAI, California, USA). Materials and methods were adapted from McCavitt et al. [18]. The 19 recommendations published in the AAOS Management of Hip Fractures in Older Adults CPG [20] (published December 3, 2021) were rewritten in question form requesting that ChatGPT-5 generate the treatment recommendation for each clinical scenario. This process was also applied to guidelines on Pediatric Developmental Dysplasia of the Hip (DDH) [21], Management of Rotator Cuff Injuries [22], and the Treatment of Metastatic Carcinoma and Myeloma of the Femur [23] to be queried to ChatGPT-5. These additional CPGs were added in order to broaden the scope of the study beyond hip fracture management and evaluate citation accuracy across multiple areas of orthopaedic practice.

This study involved no human subjects or protected health information; therefore, institutional review board approval was not required.

Prompt generation

**Figure 1:**
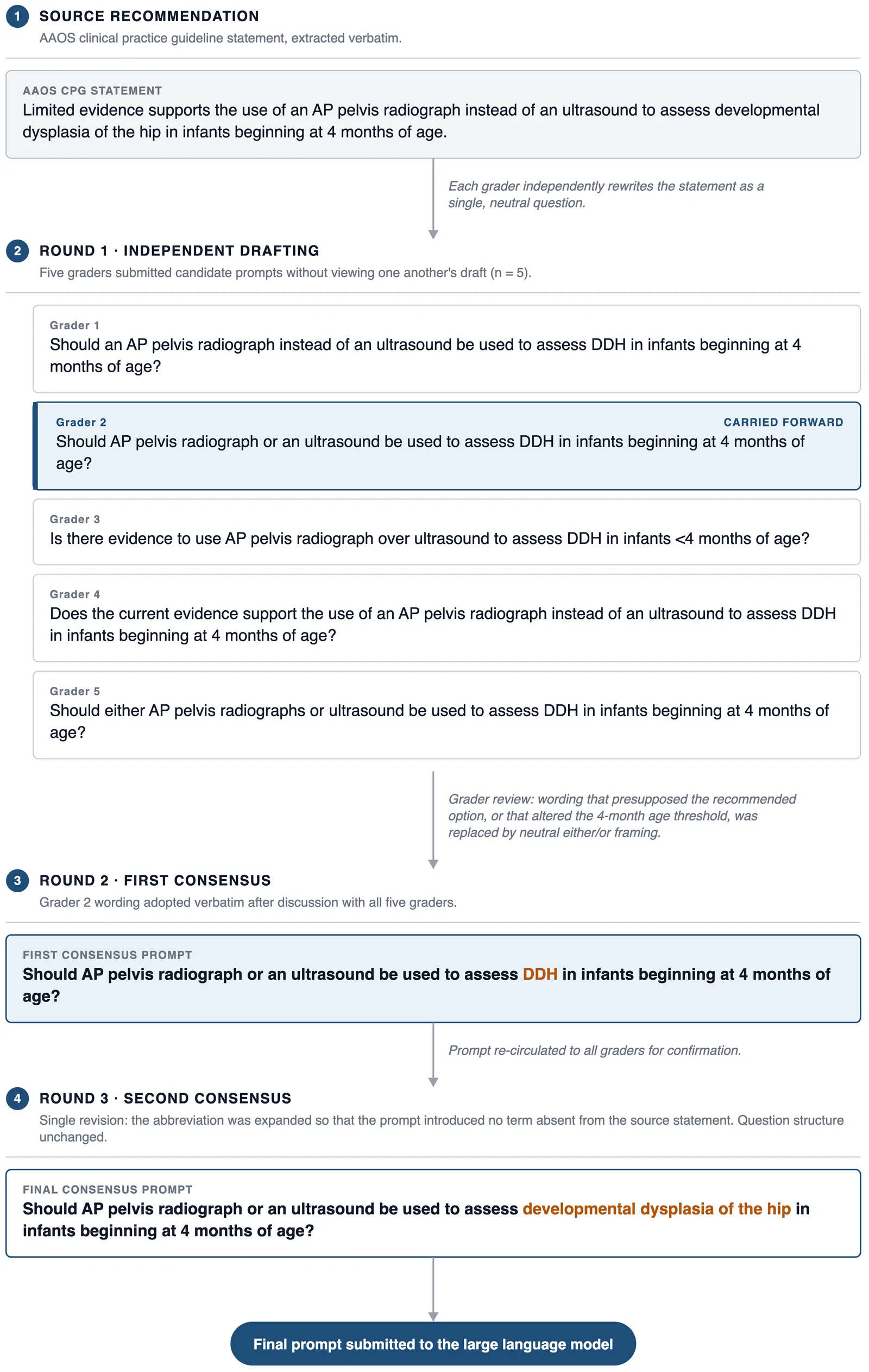
Example of the internal consensus process used to generate prompts. Five reviewers drafted a version of the American Academy of Orthopaedic Surgeons (AAOS) Clinical Practice Guidelines (CPGs) rewritten in question form. After discussion, the preferred prompt version was carried forward and revised until unanimous agreement was achieved.

A structured internal consensus process was used to generate standardized prompts that accurately reflected the AAOS CPG recommendations. Each of the five medical student graders independently drafted prompts based on the individual recommendations. Differences in wording were discussed, and the prompts were revised until all five graders reached unanimous agreement. Three rounds of prompt refinement were performed. The finalized prompts were reviewed and approved by the senior author before data collection. An example illustrating the initial prompt submissions and subsequent consensus process is provided in Figure 1. Before evaluating the generated citations, all five graders reviewed and discussed each CPG to establish familiarity with its content. All finalized prompts are provided in Appendix A.

Data collection

Responses and supporting evidence were collected by five independent graders. Each grader queried ChatGPT-5 using all 70 standardized prompts, resulting in 350 independently generated responses. Because ChatGPT can produce different responses to the same prompt, each grader evaluated only the citations generated in their own responses; graders did not independently rate a shared set of citations. A new conversation thread was initiated for each CPG, and each grader used a separate paid ChatGPT account. Web search was available and could be invoked automatically to reflect how users may request medical information with supporting references. The investigators did not control whether it was used for each query. The search provider, number of retrieved results, retrieval logic, and integration of retrieved information into the generated responses were not displayed to or controlled by the investigators. No browsing-disabled condition was evaluated. Each generated reference was compared with its corresponding PubMed record, and any discrepancy in an evaluated citation element was recorded as incorrect. The total number of citation errors was collected for citation accuracy data by aggregating the data collected by all five evaluators. Graders were blinded to the responses and assessments of the other graders during data collection.

Assessment and statistics

Data on the citations were collected as assessed by each independent evaluator for all 70 recommendations. Citation information evaluated included the presence of hallucination, title, author, journal, year, pages, volume, and PMID. Hallucination was defined as the citation of nonexistent studies, studies not indexed in PubMed, or with PMIDs not associated with a published study. Inaccuracies in citation elements were defined as any discrepancy between the proper citation elements listed in PubMed [18]. Comparisons to previously published ChatGPT-4o mini data were exploratory and intended to provide historical context rather than establish causal model superiority, given unavailable prompt-level replication. Citation-level results were aggregated across the 350 independently generated responses. Because each grader evaluated the unique citations generated in their own responses rather than independently rating a shared citation set, inter-rater reliability statistics and disagreement adjudication were not applicable to the collected dataset. Descriptive statistics were computed using spreadsheet software (Google Sheets; Google LLC, Mountain View, CA, USA).

A reference was classified as fully correct only when all seven evaluated bibliographic elements matched the corresponding PubMed record. A reference with any incorrect, missing, or fabricated element was not classified as fully correct. The fully correct reference rate was calculated as the number of fully correct references divided by the total number of generated references. A query was classified as fully correct only when every reference generated in that response was fully correct; the fully correct query rate was calculated as the number of fully correct queries divided by the 350 total queries. The complete query-level analytic dataset is available for reference in Appendix B. Each row represents one independently generated response and includes the CPG, recommendation number, total references generated, counts of fabricated references and errors in each citation element, number of fully correct references, and whether all references within the query were fully correct.

## Results

A total of 350 ChatGPT queries were performed by five graders across 70 orthopaedic recommendations, generating 2,736 references. Of these, 19 recommendations pertained to fractures (hip fractures), 29 to sports medicine (rotator cuff injuries), nine to pediatrics (DDH), and 13 to orthopaedic oncology. Overall, 7.13% of references were hallucinated or fabricated (n = 195). Citation inaccuracies varied in frequency across all subspecialties. Among individual citation elements, overall error rates were highest for PMID (n = 1,020, 37.28%), title (n = 840, 30.70%), and pages (n = 752, 27.49%), followed by volume (n = 700, 25.58%), journal (n = 517, 18.90%), author (n = 444, 16.23%), and year (n = 198, 7.24%). Only 1,350 references (49.34%) were fully correct with no citation element errors, and all citations within a single query were entirely accurate in just 28 (8.00%) cases. A full breakdown of the citation accuracy counts by subspecialty evaluated and aggregated totals is available in Appendix C. 

When evaluated per-query, fabricated references and incorrect publication years were absent in the majority of responses (median 0, IQR 0-1 for both), while incorrect PMIDs were the most frequent error (median 3, IQR 0-5). Because per-query error counts were right-skewed and zero-inflated, ChatGPT-5 citation metrics are summarized as median (Q1-Q3) (Table 1).

**Table 1:** Per-query median, interquartile ranges, and total range for each citation element.

Citation metric	Median (Q1–Q3)	Range
Fabricated references	0 (0–1)	0–7
References	8 (6–9)	3–18
Title incorrect	2 (1–4)	0–10
Author incorrect	1 (0–2)	0–8
Journal incorrect	1 (0–2)	0–8
Year incorrect	0 (0–1)	0–7
Pages incorrect	2 (0–3.75)	0–10
Volume incorrect	2 (0–3)	0–10
PMID incorrect	3 (0–5)	0–15

## Discussion

The default, user-facing version of ChatGPT-5 demonstrated variable accuracy across all evaluated citation components, including title, authorship, year, journal, volume, pages, and PMID. Fewer than half of the citations produced by the ChatGPT system were entirely correct, indicating that at least one of the seven evaluated citation components was incorrect or missing in more than half of the generated references. Only 8% of evaluated queries contained exclusively fully correct citations based on PubMed metadata. Among queries derived from the DDH CPG, none contained exclusively fully correct citations. Together, these findings demonstrate that citation results outputted by the evaluated system should be approached with a level of scrutiny and require independent bibliographic verification at this time.

McCavitt et al. [18] previously reported that ChatGPT-4o mini generated inaccurate and fabricated citations in response to prompts based on the AAOS Hip Fracture CPG. Across 2,556 references, the authors reported a fabrication rate of 7.9%, while studies of earlier model versions reported rates ranging from 18% to 39.6% [18]. In the present study, ChatGPT-5 produced 195 fabricated references among 2,736 total references, corresponding to a fabrication rate of 7.13%. Although the proportion observed in the present study was numerically lower, the studies differed in prompts, CPGs evaluated, accounts, and browsing conditions. The values therefore provide historical context but should not be interpreted as evidence that ChatGPT-5 performed better than ChatGPT-4o mini. Regardless of these cross-study differences, fabricated references persisted in the evaluated system and require independent verification. 

Error patterns

Several patterns were observed among the citation errors generated by ChatGPT-5. Titles were frequently truncated or blended with the titles of similarly named studies. When multiple studies had similar titles, ChatGPT occasionally combined elements from separate publications into a single title. Author lists frequently contained omitted authors, fabricated names, or researchers associated with related studies. PMIDs were occasionally incorrect by only one or several digits, entirely fabricated, reported as unavailable, or linked to a different article within the same journal or volume. Errors also clustered across related bibliographic fields. For example, when the journal was incorrect, the associated volume and page numbers were often incorrect as well, suggesting linked citation-reconstruction errors rather than isolated mistakes within individual metadata fields. Citation accuracy also appeared lower for studies with overlapping author groups, publication years, journals, or closely related titles. By contrast, well-established studies and publications with more distinctive titles, authorship groups, and publication sources appeared more likely to be cited correctly. Prior studies suggest that PMID errors may reflect limitations in the model’s processing or reconstruction of digital object identifiers [13]. These errors remained common in the present study, indicating a continued need for more reliable bibliographic verification.

These patterns are consistent with proposed explanations for LLM-generated reference hallucinations. Bhattacharyya et al. [13] suggested that LLMs generate text by predicting likely subsequent tokens based on statistical patterns learned during model training rather than by directly retrieving verified bibliographic records. Sanchez-Ramos et al. [24] additionally proposed that inaccuracies or inconsistencies within the underlying training data may contribute to reference hallucinations. Bhattacharyya et al. [13] further suggested that PMID inaccuracies may be related to limitations in the model’s processing or reconstruction of digital object identifiers. The present study did not investigate the mechanisms responsible for the observed errors. Nevertheless, their frequency demonstrates that references generated by the evaluated system require verification before clinical or academic use.

Retrieval-Augmented Generation (RAG)

Medical-informatics systems increasingly incorporate RAG to mitigate hallucinations. RAG supplements an LLM by retrieving information relevant to the user’s query from an external database or document collection before generating a response. This approach represents a promising strategy for reducing hallucinations and limiting the propagation of unsupported medical information. One recent study demonstrated a substantial reduction in hallucinations when responses were grounded in a curated RAG database, although this improvement was accompanied by a lower answer rate because the system more frequently abstained when relevant information could not be retrieved [25]. Accordingly, the present findings should be interpreted as a descriptive benchmark of the general-purpose ChatGPT system’s citation accuracy rather than the performance of either an isolated language model or a purpose-built clinical retrieval system. Although general-purpose citation generation may not represent current best practice for clinical reference verification, it remains a clinically relevant use case because users may interact with general-purpose LLMs without access to, or awareness of, a curated retrieval pipeline. RAG could reduce the error rates observed in this study; however, its effectiveness remains dependent on the quality, completeness, and relevance of the underlying knowledge base, as well as the accuracy of the retrieval and generation processes.

Limitations

The present study evaluated citation accuracy based on discrepancies between generated bibliographic metadata and the corresponding PubMed record. However, an LLM may also hallucinate the relevance or evidentiary support of a citation. A citation may contain entirely correct bibliographic metadata while referring to a study that is irrelevant to the query or does not support the associated clinical claim. Because this study did not evaluate whether each cited publication was clinically relevant to the corresponding recommendation, the reported fully correct citation rate of 49.34% should be interpreted as an upper bound on citation reliability rather than as an estimate of clinically usable citation support. Citation accuracy was intentionally evaluated as a binary bibliographic outcome: a citation element either matched the corresponding PubMed record or did not. The study did not separately classify the magnitude or practical consequences of inaccuracies; therefore, errors ranging from minor typographical discrepancies to substantially inaccurate bibliographic information contributed equally to the element-level error counts.

Each generated response and its citations were evaluated by a single medical student grader rather than independently verified by multiple graders, an orthopaedic surgeon, or a medical librarian. Because graders evaluated citations from their own independently generated responses rather than a shared citation set, inter-rater reliability could not be calculated. The absence of duplicate citation-level verification introduces the possibility of classification or data-entry errors.

Responses may have been influenced by prior interactions, conversational context, personalization features, or account-specific settings associated with each grader’s ChatGPT account. Although the use of multiple graders may have reduced the influence of any single user’s interaction history, account-level variation may still have affected the generated responses. Web search was available under the default settings of the user-facing ChatGPT interface and could be triggered automatically by the system. Because the investigators did not control or systematically record whether search was invoked for each query, the contribution of web retrieval to the generated citations could not be isolated, although an ablation experiment may provide further description of the model's citation generation capabilities. Accordingly, this study evaluated the default user-facing interface of ChatGPT-5 rather than either an isolated language model or a consistently browsing-enabled system. Model parameters such as the exact model snapshot, temperature, top-p, and maximum token limit were also not displayed or controlled through the user-facing interface, limiting strict replication. In addition, while contextual comparisons to McCavitt et al.’s evaluation of ChatGPT-4o mini were made, differences in prompts, CPG coverage, accounts, system configurations, and data-collection procedures limit direct comparability [18]. Comparisons between models should therefore be interpreted as contextual rather than definitive head-to-head evaluations.

The data were hierarchically structured, with references nested within queries and queries nested within CPG recommendations. The primary reference-level proportions give greater weight to queries that generated more references and should not be interpreted as query-level or recommendation-level estimates. Because this investigation was designed as a descriptive study, no inferential comparisons were performed; future inferential studies should account for clustering using mixed-effects models or generalized estimating equations. The study protocol and statistical analysis plan were not preregistered, although the complete query-level analytic dataset has been provided to permit independent reproduction of the reported descriptive results. Finally, this study only evaluated queries based on four AAOS guidelines, limiting generalizability to other orthopaedic topics and medical specialties.

Future directions

Future studies should evaluate both the bibliographic accuracy and clinical relevance of generated references, use duplicate independent citation verification, compare browsing-enabled and browsing-disabled conditions, and directly compare models using identical prompts and controlled system settings. Studies could also compare general-purpose systems with standardized RAG systems and analyze the severity and co-occurrence of different citation errors.

## Conclusions

Citation errors remain frequent in references generated by browsing-enabled ChatGPT-5 in response to AAOS CPG-based questions. Fewer than half of all generated references were fully correct across the seven evaluated bibliographic elements, and fabricated references were observed. Only 8% of queries contained exclusively fully correct citations. These findings describe bibliographic accuracy under the specific prompts and system configuration evaluated and should not be interpreted as evidence of comparative performance relative to other models although may provide a benchmark for future work. References generated by ChatGPT should be independently verified against authoritative bibliographic sources before academic or clinical use.

## Appendices

Appendix A 

**Table 2:** Query-level dataset. One line per query with the recommendation category, number, total number of references, and citation factors incorrect. "All correct in query" is reported in binary.

Query	CPG category	Recommendation number	Number of fabricated references	References generated	Title incorrect	Author incorrect	Journal incorrect	Year incorrect	Pages incorrect	Volume incorrect	PMID incorrect	Correct citations	All correct in query
1	Hip fracture	1	0	5	1	1	1	0	2	2	0	3	0
2	Hip fracture	1	0	8	0	0	1	0	0	0	0	7	0
3	Hip fracture	1	0	8	3	1	1	1	0	0	1	5	0
4	Hip fracture	1	0	9	2	0	0	0	0	0	0	7	0
5	Hip fracture	1	0	10	3	1	4	0	3	2	2	6	0
6	Hip fracture	2	0	6	2	1	0	0	2	2	1	4	0
7	Hip fracture	2	0	7	1	0	2	0	2	2	0	5	0
8	Hip fracture	2	0	6	1	0	1	0	1	1	1	5	0
9	Hip fracture	2	0	9	1	0	0	0	0	0	1	8	0
10	Hip fracture	2	0	7	3	1	1	0	1	0	2	4	0
11	Hip fracture	3	0	6	0	0	0	0	0	0	3	3	0
12	Hip fracture	3	0	8	0	0	0	0	0	0	0	8	1
13	Hip fracture	3	1	9	5	2	4	1	5	5	3	4	0
14	Hip fracture	3	0	4	0	0	0	0	0	0	0	4	1
15	Hip fracture	3	0	8	1	1	0	0	1	0	1	7	0
16	Hip fracture	4	0	10	0	0	0	0	0	0	0	10	1
17	Hip fracture	4	0	5	2	0	1	0	1	1	3	2	0
18	Hip fracture	4	1	7	4	5	2	1	6	4	3	1	0
19	Hip fracture	4	0	5	0	0	0	0	0	1	0	4	0
20	Hip fracture	4	0	9	6	2	3	0	6	4	1	3	0
21	Hip fracture	5	0	12	4	5	1	1	3	2	1	7	0
22	Hip fracture	5	0	7	1	0	1	1	1	1	3	4	0
23	Hip fracture	5	0	8	3	3	2	0	3	3	1	5	0
24	Hip fracture	5	0	10	1	3	3	0	1	3	1	7	0
25	Hip fracture	5	0	7	1	0	1	0	2	1	0	5	0
26	Hip fracture	6	0	8	4	1	1	0	4	4	7	1	0
27	Hip fracture	6	0	7	0	0	0	1	0	1	5	2	0
28	Hip fracture	6	0	6	2	1	2	0	2	2	0	4	0
29	Hip fracture	6	0	9	5	0	1	0	0	2	0	4	0
30	Hip fracture	6	1	6	3	2	1	0	1	2	2	3	0
31	Hip fracture	7	0	7	3	0	1	0	1	1	4	3	0
32	Hip fracture	7	0	6	1	0	1	0	1	1	4	2	0
33	Hip fracture	7	0	7	4	2	1	0	2	1	0	3	0
34	Hip fracture	7	2	7	2	0	0	0	0	0	4	3	0
35	Hip fracture	7	0	5	2	1	1	0	1	1	1	3	0
36	Hip fracture	8	1	7	1	2	1	0	1	1	4	3	0
37	Hip fracture	8	0	8	2	1	0	0	0	0	0	6	0
38	Hip fracture	8	1	8	3	3	2	1	2	2	2	5	0
39	Hip fracture	8	0	11	1	1	0	1	2	2	0	9	0
40	Hip fracture	8	0	8	4	2	2	0	2	2	6	4	0
41	Hip fracture	9	0	8	0	0	0	0	0	0	0	8	1
42	Hip fracture	9	0	10	0	0	0	0	0	0	0	10	1
43	Hip fracture	9	0	10	8	3	3	0	7	6	2	2	0
44	Hip fracture	9	0	5	0	0	0	0	0	0	0	5	1
45	Hip fracture	9	0	10	6	1	3	2	5	4	9	1	0
46	Hip fracture	10	1	4	1	0	0	0	0	0	3	1	0
47	Hip fracture	10	0	7	4	3	0	0	0	0	0	3	0
48	Hip fracture	10	1	8	7	4	4	2	7	6	3	1	0
49	Hip fracture	10	0	10	4	0	0	0	2	2	1	6	0
50	Hip fracture	10	0	8	6	3	1	0	6	4	2	2	0
51	Hip fracture	11	1	6	3	1	1	1	1	1	3	3	0
52	Hip fracture	11	0	11	1	4	1	0	0	0	0	7	0
53	Hip fracture	11	0	5	1	2	1	1	3	2	3	2	0
54	Hip fracture	11	0	10	6	0	0	0	0	0	0	4	0
55	Hip fracture	11	0	5	3	2	0	0	1	2	2	2	0
56	Hip fracture	12	2	5	0	0	0	0	0	0	2	3	0
57	Hip fracture	12	0	11	3	0	0	0	0	0	0	8	0
58	Hip fracture	12	1	7	3	3	1	1	2	2	2	4	0
59	Hip fracture	12	1	9	0	0	0	1	0	0	8	1	0
60	Hip fracture	12	0	8	2	0	1	0	1	2	3	5	0
61	Hip fracture	13	0	3	0	0	0	0	0	0	0	3	1
62	Hip fracture	13	0	4	0	0	0	0	0	0	1	3	0
63	Hip fracture	13	1	6	2	4	1	2	2	2	2	2	0
64	Hip fracture	13	0	6	2	0	2	0	0	1	0	4	0
65	Hip fracture	13	0	6	2	2	2	0	2	2	3	3	0
66	Hip fracture	14	0	7	4	1	5	0	6	5	1	1	0
67	Hip fracture	14	0	12	3	0	2	0	0	0	0	3	0
68	Hip fracture	14	0	6	1	1	0	0	2	1	6	0	0
69	Hip fracture	14	1	7	3	0	1	1	1	1	5	2	0
70	Hip fracture	14	0	10	3	3	4	1	6	6	6	4	0
71	Hip fracture	15	0	13	0	0	0	0	0	0	0	13	1
72	Hip fracture	15	0	7	2	0	2	0	1	1	6	1	0
73	Hip fracture	15	2	9	4	5	5	2	5	5	5	4	0
74	Hip fracture	15	0	10	7	7	2	0	7	8	0	2	0
75	Hip fracture	15	0	10	7	8	6	0	7	5	1	2	0
76	Hip fracture	16	0	5	1	2	0	0	0	1	2	3	0
77	Hip fracture	16	2	8	1	0	0	0	0	0	4	4	0
78	Hip fracture	16	0	6	0	2	0	0	2	0	0	4	0
79	Hip fracture	16	1	6	1	1	0	0	0	0	3	3	0
80	Hip fracture	16	0	8	5	2	4	0	5	5	4	3	0
81	Hip fracture	17	0	5	1	2	0	0	0	1	2	3	0
82	Hip fracture	17	0	13	1	0	0	0	0	0	0	12	0
83	Hip fracture	17	0	8	5	3	1	0	4	5	1	3	0
84	Hip fracture	17	1	10	3	3	1	0	2	2	6	4	0
85	Hip fracture	17	0	7	2	1	1	0	1	1	3	4	0
86	Hip fracture	18	0	7	0	0	0	0	0	0	0	7	1
87	Hip fracture	18	0	14	0	0	0	0	0	0	0	14	1
88	Hip fracture	18	4	6	5	4	4	4	5	4	5	1	0
89	Hip fracture	18	3	7	2	0	0	1	0	0	3	4	0
90	Hip fracture	18	0	7	2	0	1	1	3	1	2	4	0
91	Hip fracture	19	0	8	0	1	0	0	0	0	0	7	0
92	Hip fracture	19	1	5	1	0	1	0	1	1	1	4	0
93	Hip fracture	19	0	5	2	1	2	0	4	4	3	1	0
94	Hip fracture	19	3	7	2	0	1	0	1	0	3	4	0
95	Hip fracture	19	0	6	2	1	3	0	4	3	4	2	0
96	Rotator cuff	1	0	8	0	0	0	0	0	0	0	8	1
97	Rotator cuff	1	0	8	3	1	1	0	2	2	1	5	0
98	Rotator cuff	1	2	8	5	5	2	2	4	4	3	3	0
99	Rotator cuff	1	0	8	3	0	0	0	2	2	0	5	0
100	Rotator cuff	1	0	7	5	1	1	2	3	3	4	2	0
101	Rotator cuff	2	0	10	2	0	1	0	1	1	4	6	0
102	Rotator cuff	2	0	6	1	0	0	0	2	2	0	4	0
103	Rotator cuff	2	0	12	3	1	1	0	1	1	5	7	0
104	Rotator cuff	2	0	6	1	1	1	0	2	2	1	4	0
105	Rotator cuff	2	1	8	2	1	1	2	2	2	6	2	0
106	Rotator cuff	3	0	5	0	0	2	0	0	0	0	3	0
107	Rotator cuff	3	0	5	0	0	0	0	0	0	0	5	1
108	Rotator cuff	3	1	7	5	3	3	1	6	6	5	1	0
109	Rotator cuff	3	0	7	3	0	0	0	2	2	2	4	0
110	Rotator cuff	3	0	12	8	4	3	0	10	9	8	2	0
111	Rotator cuff	4	0	6	1	1	1	0	2	2	1	4	0
112	Rotator cuff	4	0	10	0	0	1	0	3	2	0	7	0
113	Rotator cuff	4	0	12	3	0	3	1	7	7	0	5	0
114	Rotator cuff	4	2	8	4	1	1	1	2	2	2	4	0
115	Rotator cuff	4	0	9	7	4	7	3	7	6	5	2	0
116	Rotator cuff	5	0	5	0	1	2	0	1	1	0	3	0
117	Rotator cuff	5	0	5	0	0	0	0	0	0	0	5	1
118	Rotator cuff	5	1	7	2	1	1	1	1	1	4	3	0
119	Rotator cuff	5	0	7	1	0	0	0	0	0	4	3	0
120	Rotator cuff	5	0	8	1	2	2	0	2	2	3	5	0
121	Rotator cuff	6	0	7	2	2	0	0	1	1	1	5	0
122	Rotator cuff	6	0	7	0	0	0	0	0	0	0	7	1
123	Rotator cuff	6	2	11	5	2	3	2	3	3	8	3	0
124	Rotator cuff	6	2	9	1	1	0	0	0	0	2	7	0
125	Rotator cuff	6	0	11	6	3	2	2	2	2	7	4	0
126	Rotator cuff	7	0	12	0	0	0	0	0	0	0	12	1
127	Rotator cuff	7	0	6	0	0	0	0	0	0	0	6	1
128	Rotator cuff	7	1	8	2	2	4	1	6	6	3	2	0
129	Rotator cuff	7	3	10	4	0	3	1	3	3	4	6	0
130	Rotator cuff	7	0	12	3	3	2	0	4	3	4	8	0
131	Rotator cuff	8	0	8	0	1	0	0	0	0	0	7	0
132	Rotator cuff	8	0	7	0	1	0	0	0	0	0	6	0
133	Rotator cuff	8	0	8	1	0	3	0	2	3	7	1	0
134	Rotator cuff	8	1	11	5	0	5	2	5	5	7	4	0
135	Rotator cuff	8	0	11	4	3	5	0	7	7	2	4	0
136	Rotator cuff	9	0	5	0	0	1	0	0	0	0	4	0
137	Rotator cuff	9	0	3	0	0	0	0	0	0	0	3	1
138	Rotator cuff	9	5	9	6	5	7	5	7	7	9	0	0
139	Rotator cuff	9	3	7	1	1	0	1	1	1	2	5	0
140	Rotator cuff	9	0	7	3	3	2	2	3	3	7	0	0
141	Rotator cuff	10	0	11	5	1	0	1	0	0	0	6	0
142	Rotator cuff	10	0	7	0	0	0	0	0	0	0	7	1
143	Rotator cuff	10	3	10	4	4	4	3	4	4	7	3	0
144	Rotator cuff	10	3	11	0	2	1	0	0	0	2	9	0
145	Rotator cuff	10	0	6	2	1	2	0	4	2	4	2	0
146	Rotator cuff	11	0	6	1	1	0	0	0	0	0	5	0
147	Rotator cuff	11	0	7	0	0	0	0	1	1	0	6	0
148	Rotator cuff	11	1	6	2	1	3	2	3	3	5	1	0
149	Rotator cuff	11	3	9	1	0	1	1	1	1	3	6	0
150	Rotator cuff	11	0	8	3	3	3	0	5	4	0	3	0
151	Rotator cuff	12	0	7	4	0	2	1	3	2	0	3	0
152	Rotator cuff	12	0	9	0	0	0	0	1	0	0	8	0
153	Rotator cuff	12	4	8	6	4	5	4	5	5	7	1	0
154	Rotator cuff	12	5	10	1	0	1	0	1	1	5	5	0
155	Rotator cuff	12	0	8	7	4	6	5	7	7	8	0	0
156	Rotator cuff	13	2	4	0	0	0	0	0	0	2	2	0
157	Rotator cuff	13	0	10	0	0	0	0	0	0	0	10	1
158	Rotator cuff	13	3	8	6	4	3	3	4	4	8	0	0
159	Rotator cuff	13	6	12	1	0	2	0	2	2	2	10	0
160	Rotator cuff	13	0	9	2	3	2	0	4	3	6	3	0
161	Rotator cuff	14	1	10	5	6	6	3	7	7	6	4	0
162	Rotator cuff	14	0	15	2	0	0	0	0	1	0	13	0
163	Rotator cuff	14	4	10	6	5	6	5	6	6	10	0	0
164	Rotator cuff	14	7	11	1	1	0	0	0	0	2	9	0
165	Rotator cuff	14	0	6	2	0	0	0	0	0	4	2	0
166	Rotator cuff	15	0	10	1	0	0	0	0	0	0	9	0
167	Rotator cuff	15	0	8	3	1	2	0	2	3	0	5	0
168	Rotator cuff	15	0	10	2	1	4	0	8	8	3	2	0
169	Rotator cuff	15	1	11	2	0	2	2	5	5	8	3	0
170	Rotator cuff	15	0	10	5	1	5	0	5	5	0	5	0
171	Rotator cuff	16	0	9	1	0	1	0	0	0	0	8	0
172	Rotator cuff	16	0	17	3	4	1	0	2	2	2	13	0
173	Rotator cuff	16	0	10	5	2	5	0	6	6	1	4	0
174	Rotator cuff	16	4	12	4	0	3	0	3	3	7	5	0
175	Rotator cuff	16	0	18	8	7	4	2	4	4	0	1	0
176	Rotator cuff	17	0	7	5	4	5	0	4	3	0	2	0
177	Rotator cuff	17	0	8	2	2	2	0	4	4	1	4	0
178	Rotator cuff	17	6	10	7	6	8	7	8	8	8	2	0
179	Rotator cuff	17	2	7	1	0	1	0	2	1	3	4	0
180	Rotator cuff	17	0	8	6	7	5	3	6	5	4	1	0
181	Rotator cuff	18	0	8	1	1	0	0	1	0	0	7	0
182	Rotator cuff	18	3	10	3	3	3	3	3	3	9	1	0
183	Rotator cuff	18	0	10	5	0	4	1	6	7	3	3	0
184	Rotator cuff	18	4	11	3	0	0	0	0	0	4	7	0
185	Rotator cuff	18	0	9	5	0	1	0	5	2	5	4	0
186	Rotator cuff	19	0	6	2	0	0	0	0	0	0	4	0
187	Rotator cuff	19	0	5	3	0	2	0	3	3	0	2	0
188	Rotator cuff	19	2	10	7	2	6	2	7	8	4	2	0
189	Rotator cuff	19	2	8	3	1	1	0	2	2	5	3	0
190	Rotator cuff	19	0	10	8	7	7	6	8	8	9	1	0
191	Rotator cuff	20	0	8	0	0	0	0	0	0	0	8	1
192	Rotator cuff	20	0	11	0	0	1	0	0	0	0	10	0
193	Rotator cuff	20	1	8	3	1	4	1	6	6	5	2	0
194	Rotator cuff	20	1	11	1	0	1	1	1	1	1	10	0
195	Rotator cuff	20	0	10	6	3	5	1	6	5	4	4	0
196	Rotator cuff	21	0	14	0	0	0	0	0	0	0	14	1
197	Rotator cuff	21	4	8	5	4	3	4	4	4	5	3	0
198	Rotator cuff	21	1	12	10	2	6	2	9	10	9	2	0
199	Rotator cuff	21	6	14	5	1	4	2	4	4	7	7	0
200	Rotator cuff	21	0	10	4	5	5	0	7	5	1	3	0
201	Rotator cuff	22	0	7	4	1	1	0	0	0	0	3	0
202	Rotator cuff	22	0	4	3	0	0	1	1	1	3	1	0
203	Rotator cuff	22	1	6	4	1	2	1	4	4	2	2	0
204	Rotator cuff	22	2	10	3	0	1	0	1	1	6	4	0
205	Rotator cuff	22	1	6	2	3	1	1	2	1	5	1	0
206	Rotator cuff	23	0	8	0	0	0	0	0	0	0	8	1
207	Rotator cuff	23	0	6	0	2	0	0	0	0	2	4	0
208	Rotator cuff	23	0	9	4	0	5	0	6	7	1	2	0
209	Rotator cuff	23	0	10	6	0	4	2	5	4	8	2	0
210	Rotator cuff	23	0	5	3	4	4	3	4	4	5	0	0
211	Rotator cuff	24	1	8	4	3	3	2	5	5	6	2	0
212	Rotator cuff	24	1	5	4	3	3	3	3	3	5	0	0
213	Rotator cuff	24	3	9	5	4	5	4	5	5	6	3	0
214	Rotator cuff	24	3	11	2	1	2	3	4	4	6	5	0
215	Rotator cuff	24	0	6	3	1	2	0	4	2	3	3	0
216	Rotator cuff	25	0	8	0	2	0	0	0	0	0	6	0
217	Rotator cuff	25	0	4	2	0	0	0	0	0	2	2	0
218	Rotator cuff	25	1	9	5	1	3	1	3	3	8	1	0
219	Rotator cuff	25	2	10	3	0	0	0	0	1	6	4	0
220	Rotator cuff	25	0	7	4	3	3	0	4	4	1	3	0
221	Rotator cuff	26	0	13	0	0	0	0	0	0	0	13	1
222	Rotator cuff	26	0	9	0	1	0	0	0	0	1	8	0
223	Rotator cuff	26	0	8	3	0	2	0	2	2	7	1	0
224	Rotator cuff	26	4	15	6	0	0	0	0	0	7	8	0
225	Rotator cuff	26	0	18	8	1	4	1	4	4	15	3	0
226	Rotator cuff	27	0	7	1	0	0	0	0	0	0	6	0
227	Rotator cuff	27	0	4	1	1	2	0	1	2	2	2	0
228	Rotator cuff	27	0	8	1	1	1	0	1	1	4	4	0
229	Rotator cuff	27	0	10	0	0	1	2	2	3	6	4	0
230	Rotator cuff	27	0	6	2	0	0	0	3	0	0	3	0
231	Rotator cuff	28	0	7	4	1	3	0	3	2	0	3	0
232	Rotator cuff	28	0	4	1	0	0	0	2	2	1	2	0
233	Rotator cuff	28	0	5	0	0	1	0	1	1	5	0	0
234	Rotator cuff	28	0	10	0	1	0	0	0	0	8	2	0
235	Rotator cuff	28	0	8	3	3	2	1	4	3	4	4	0
236	Rotator cuff	29	0	9	0	0	0	0	0	0	0	9	1
237	Rotator cuff	29	0	5	2	2	1	0	3	2	1	2	0
238	Rotator cuff	29	1	3	1	1	1	1	2	2	2	1	0
239	Rotator cuff	29	2	9	1	0	0	0	1	1	6	3	0
240	Rotator cuff	29	0	10	2	5	2	0	5	2	4	5	0
241	DDH	1	0	12	3	5	2	1	2	1	0	7	0
242	DDH	1	0	5	0	0	0	0	0	0	3	2	0
243	DDH	1	1	8	3	2	2	1	1	2	5	3	0
244	DDH	1	2	8	1	0	0	0	1	0	2	6	0
245	DDH	1	0	8	1	0	1	2	2	2	6	2	0
246	DDH	2	0	8	2	0	1	0	0	0	0	6	0
247	DDH	2	0	4	2	0	0	0	0	1	2	2	0
248	DDH	2	0	7	1	0	2	1	2	3	3	4	0
249	DDH	2	0	7	2	0	1	0	1	1	4	3	0
250	DDH	2	0	5	1	1	1	1	1	1	4	1	0
251	DDH	3	0	6	0	1	0	0	0	0	0	5	0
252	DDH	3	1	4	2	1	1	2	2	2	3	1	0
253	DDH	3	0	7	4	1	4	1	4	4	5	2	0
254	DDH	3	1	8	2	1	1	1	1	1	4	4	0
255	DDH	3	0	8	7	2	3	0	4	3	7	1	0
256	DDH	4	0	7	0	1	0	0	0	0	0	6	0
257	DDH	4	0	4	2	1	2	0	2	2	3	1	0
258	DDH	4	0	5	0	0	0	0	0	0	3	2	0
259	DDH	4	0	5	0	1	0	1	1	1	3	2	0
260	DDH	4	0	6	3	1	1	0	3	1	2	3	0
261	DDH	5	0	6	1	0	2	0	1	1	0	4	0
262	DDH	5	0	4	1	0	0	1	0	0	2	2	0
263	DDH	5	0	5	1	1	0	0	1	1	3	2	0
264	DDH	5	0	5	0	0	0	0	0	0	3	2	0
265	DDH	5	0	4	1	0	0	0	0	0	3	1	0
266	DDH	6	0	9	0	2	0	0	0	0	0	7	0
267	DDH	6	0	4	2	1	1	0	1	2	2	2	0
268	DDH	6	1	5	1	3	2	2	3	3	5	0	0
269	DDH	6	0	8	5	0	0	0	6	5	2	2	0
270	DDH	6	0	6	5	4	1	0	3	1	4	1	0
271	DDH	7	0	4	1	2	2	0	2	2	1	2	0
272	DDH	7	0	4	1	0	0	0	1	1	1	3	0
273	DDH	7	0	6	1	0	0	0	1	1	4	2	0
274	DDH	7	0	8	2	0	0	0	0	0	4	4	0
275	DDH	7	0	8	0	1	1	1	1	1	7	1	0
276	DDH	8	0	8	1	3	0	0	0	0	0	5	0
277	DDH	8	0	4	0	2	1	0	1	1	3	1	0
278	DDH	8	0	6	4	0	2	1	3	3	3	3	0
279	DDH	8	0	6	5	0	4	0	6	6	6	0	0
280	DDH	8	0	7	5	3	3	0	6	6	6	1	0
281	DDH	9	0	9	1	0	1	0	1	0	1	8	0
282	DDH	9	0	5	0	2	0	0	2	2	5	0	0
283	DDH	9	1	8	3	2	2	2	2	2	7	1	0
284	DDH	9	0	6	0	0	0	0	0	0	2	4	0
285	DDH	9	0	7	3	2	3	2	3	3	6	1	0
286	Oncology	1	0	7	0	0	0	0	0	0	0	7	1
287	Oncology	1	0	5	1	0	1	0	1	1	4	1	0
288	Oncology	1	1	7	4	2	2	2	4	4	3	3	0
289	Oncology	1	3	13	3	0	3	1	3	3	8	5	0
290	Oncology	1	0	7	2	0	2	0	3	2	6	1	0
291	Oncology	2	0	9	0	1	0	0	0	0	0	8	0
292	Oncology	2	1	6	3	1	2	2	2	2	2	3	0
293	Oncology	2	0	9	2	1	0	0	0	1	0	7	0
294	Oncology	2	0	8	7	0	1	1	3	3	3	1	0
295	Oncology	2	0	9	4	3	3	1	5	3	6	3	0
296	Oncology	3	0	5	1	0	0	0	2	1	0	3	0
297	Oncology	3	0	6	1	0	0	0	0	0	2	4	0
298	Oncology	3	0	7	1	0	0	0	0	0	0	6	0
299	Oncology	3	0	9	4	1	2	0	2	2	4	5	0
300	Oncology	3	0	8	7	2	2	0	2	2	4	1	0
301	Oncology	4	0	11	0	0	0	0	0	0	0	11	1
302	Oncology	4	0	5	3	1	0	0	1	1	3	2	0
303	Oncology	4	0	8	2	0	0	1	1	1	1	6	0
304	Oncology	4	0	10	8	0	0	0	0	0	2	2	0
305	Oncology	4	0	8	5	1	0	0	0	0	1	3	0
306	Oncology	5	0	7	0	0	0	0	0	0	3	4	0
307	Oncology	5	0	5	3	0	1	0	4	3	3	1	0
308	Oncology	5	2	10	5	5	6	2	7	7	6	3	0
309	Oncology	5	1	10	1	0	0	0	0	0	6	4	0
310	Oncology	5	0	10	1	0	2	1	2	2	6	4	0
311	Oncology	6	0	7	0	0	0	0	0	0	0	7	1
312	Oncology	6	0	5	3	2	2	0	4	3	3	1	0
313	Oncology	6	1	7	4	6	3	1	4	5	4	1	0
314	Oncology	6	1	8	2	1	2	0	3	2	3	5	0
315	Oncology	6	0	7	4	3	2	0	3	3	5	2	0
316	Oncology	7	0	7	1	1	1	0	0	0	0	6	0
317	Oncology	7	0	6	5	0	2	0	5	4	4	1	0
318	Oncology	7	0	7	0	4	1	0	4	4	3	3	0
319	Oncology	7	3	11	1	0	0	0	1	1	4	7	0
320	Oncology	7	0	6	1	1	1	1	3	3	5	1	0
321	Oncology	8	3	7	0	0	0	0	0	0	2	5	0
322	Oncology	8	0	6	2	0	0	0	2	3	5	1	0
323	Oncology	8	1	7	4	2	3	1	4	4	5	2	0
324	Oncology	8	0	7	0	0	1	1	1	1	3	4	0
325	Oncology	8	0	6	2	3	5	3	5	5	5	1	0
326	Oncology	9	0	6	1	0	1	0	2	2	1	4	0
327	Oncology	9	0	5	3	1	0	0	1	2	1	2	0
328	Oncology	9	1	11	6	4	3	2	3	3	6	5	0
329	Oncology	9	0	10	3	0	1	2	3	2	7	3	0
330	Oncology	9	0	6	3	0	0	0	2	0	4	2	0
331	Oncology	10	0	6	3	0	0	0	2	0	0	3	0
332	Oncology	10	0	4	3	0	0	0	3	3	3	1	0
333	Oncology	10	0	8	1	2	0	0	1	2	7	1	0
334	Oncology	10	0	12	5	0	4	1	4	4	11	1	0
335	Oncology	10	0	9	3	1	2	0	3	2	9	0	0
336	Oncology	11	0	4	0	0	0	0	0	0	0	4	1
337	Oncology	11	0	5	1	1	1	0	1	2	4	1	0
338	Oncology	11	0	5	1	0	1	0	2	2	2	3	0
339	Oncology	11	3	8	1	0	0	0	0	0	5	3	0
340	Oncology	11	0	8	2	1	1	1	2	1	7	1	0
341	Oncology	12	0	10	0	0	0	0	1	0	0	9	0
342	Oncology	12	0	5	1	1	0	1	3	2	3	2	0
343	Oncology	12	1	7	3	7	2	1	5	5	6	0	0
344	Oncology	12	5	9	0	0	0	0	0	0	3	6	0
345	Oncology	12	0	6	3	2	3	1	3	3	6	0	0
346	Oncology	13	1	8	1	0	1	0	0	0	1	7	0
347	Oncology	13	0	6	3	4	3	1	4	5	4	2	0
348	Oncology	13	1	8	4	4	5	1	5	5	3	3	0
349	Oncology	13	1	11	2	1	1	1	1	1	9	2	0
350	Oncology	13	0	6	4	1	3	0	4	3	5	1	0

Appendix B 

**Table 3:** All query prompts utilized during data collection.

Original CPG	General
Hip Fracture	
1. Preoperative traction should not routinely be used for patients with a hip fracture.	Does medical literature support the use of preoperative traction in hip fracture management?
2. Hip fracture surgery within 24-48 hours of admission may be associated with better outcomes.	Is hip fracture surgery within 24-48 hours of admission associated with better outcomes?
3. Venous thromboembolism (VTE) prophylaxis should be used in hip fracture patients.	Should venous thromboembolism (VTE) prophylaxis be used in hip fracture patients?
4. Either spinal or general anesthesia is appropriate for patients with a hip fracture.	Can either spinal or general anesthesia be used for patients with a hip fracture?
5. In patients with unstable (displaced) femoral neck fractures, arthroplasty is recommended over fixation.	Which is recommended for patients with unstable (displaced) femoral neck fractures: arthroplasty or fixation?
6. In patients with unstable (displaced) femoral neck fractures, unipolar or bipolar hemiarthroplasty can be equally beneficial.	In patients with unstable (displaced) femoral neck fractures, is unipolar or bipolar hemiarthroplasty more beneficial?
7. In properly selected patients with unstable (displaced) femoral neck fractures, there may be a functional benefit to total hip arthroplasty over hemi arthroplasty at the risk of increasing complications.	In properly selected patients with unstable (displaced) femoral neck fractures, is there a functional benefit to total hip arthroplasty over hemi arthroplasty at the risk of increasing complications?
8. In patients undergoing arthroplasty for femoral neck fractures, the use of cemented femoral stems is recommended.	In patients undergoing arthroplasty for femoral neck fractures, is the use of cemented femoral stems recommended?
9. In patients undergoing treatment of femoral neck fractures with hip arthroplasty, evidence does not show a favored surgical approach.	Does the current medical literature show a favored surgical approach in patients undergoing treatment of femoral neck fractures with hip arthroplasty?
10. In patients with stable intertrochanteric fractures, use of either a sliding hip screw or a cephalomedullary device is recommended.	Is the use of a sliding hip screw or a cephalomedullary device recommended in patients with stable intertrochanteric fractures?
11. In patients with subtrochanteric or reverse obliquity fractures a cephalomedullary device is recommended.	In patients with subtrochanteric or reverse obliquity fractures, is a cephalomedullary device recommended?
12. Patients with unstable intertrochanteric fractures should be treated with a cephalomedullary device.	Should patients with unstable intertrochanteric fractures be treated with a cephalomedullary device?
13. A blood transfusion threshold of no higher than 8g/dl is suggested in asymptomatic postoperative hip fracture patients.	Is a blood transfusion threshold of 8g/dL appropriate in asymptomatic postoperative hip fracture patients?
14. Multimodal analgesia incorporating preoperative nerve block is recommended to treat pain after hip fracture.	Is multimodal analgesia with a preoperative nerve block recommended for treating pain after a hip fracture.
15. Tranexamic acid should be administered to reduce blood loss and blood transfusion in patients with hip fractures.	Should tranexamic acid be administered to reduce blood loss and blood transfusion in patients with hip fractures?
16. Interdisciplinary care programs should be used in the care of hip fracture patients to decrease complications and improve outcomes.	Should interdisciplinary care programs be used in the care of hip fracture patients to decrease complications and improve outcomes?
17. In patients with stable (impacted/non-displaced) femoral neck fractures, hemiarthroplasty, internal fixation or non-operative care may be considered.	Should hemiarthroplasty, internal fixation, or non-operative care be considered for patients with stable (impacted/non-displaced) femoral neck fractures?
18. In patients with pertrochanteric femur fractures, short or long cephalomedullary nail may be considered.	Can short or long cephalomedullary nails be used in patients with pertrochanteric femur fractures?
19. Following surgical treatment of hip fractures, immediate, full weight bearing to tolerance may be considered.	Following surgical treatment of hip fractures, should immediate, full weight bearing to tolerance be considered?
Rotator Cuff Tear	
1. Both physical therapy and operative treatment result in significant improvement in patient-reported outcomes for patients with symptomatic small to medium fullthickness rotator cuff tears.	Do both physical therapy and operative treatment result in significant improvement in patient-reported outcomes for patients with symptomatic small to medium full thickness rotator cuff tears?
2. Patient reported outcomes (PROs) improve with physical therapy in symptomatic patients with full-thickness rotator cuff tears. However, the rotator cuff tear size, muscle atrophy, and fatty infiltration may progress over 5 to 10 years with nonoperative management.	Do patient reported outcomes (PROs) improve with physical therapy in symptomatic patients with full-thickness rotator cuff tears? Will there be progression of rotator cuff tear size, muscle atrophy, or fatty infiltration with nonoperative management?
3. Healed rotator cuff repairs show improved patient-reported and functional outcomes compared to physical therapy and unhealed rotator cuff repairs.	Do healed rotator cuff repairs show improved patient-reported and functional outcomes when compared with physical therapy and unhealed rotator cuff repairs?
4. The routine use of acromioplasty as a concomitant treatment is not suggested for therapeutic benefit as compared to arthroscopic repair alone for patients with small to medium sized full-thickness rotator cuff tears.	Is there a therapeutic benefit to the routine use of acromioplasty as a concomitant treatment compared to arthroscopic repair alone for patients with small to medium sized full-thickness rotator cuff tears?
5. Clinical examination can be useful to diagnose or stratify patients with rotator cuff tears; however, a combination of tests will increase diagnostic accuracy compared to any single clinical examination test.	Considering that clinical examination can be useful to diagnose or stratify patients with rotator cuff tears, will a combination of tests increase diagnostic accuracy compared to only utilizing a single clinical examination test?
6. MRI, MRA, CT and ultrasound are useful adjuncts to a clinical exam and radiographs for identifying rotator cuff tears.	Are MRI, MRA, CT, and ultrasound useful adjuncts to a clinical exam and radiographs for identifying rotator cuff tears?
7. Postoperative clinical and patient-reported outcomes are similar for small to medium sized full-thickness rotator cuff tears managed with early mobilization or delayed mobilization (delayed up to 8 weeks) for patients who have undergone arthroscopic rotator cuff repair.	What is the difference in postoperative clinical and patient reported outcomes in patients managed with early mobilization vs delayed mobilization (delayed up to 8 weeks) in patients who have undergone arthroscopic rotator cuff repair?
8. Following arthroscopic rotator cuff repair, in certain patient populations, outcomes are not adversely affected with immediate weaning of sling use to allow active ROM for ADLs compared to prolonged sling use because similar post-operative healing, functional outcomes, and patient-reported outcomes are achieved.	Following arthroscopic rotator cuff repair, are outcomes adversely affected with immediate weaning of sling use to allow active ROM for ADLs compared to prolonged sling use?
9. Visits of physical therapy for supervision of exercises that are performed independently at home do not provide greater improvements in pain and function outcomes (at 3 months, up to 1 year) compared to a single session of physical therapist instruction followed by an independent home program in patients following arthroscopic rotator cuff repair for small tears.	Are there any significant improvements in pain and function outcomes (at 3 months, up to 1 year) for patients following arthroscopic rotator cuff repair for small tears when comparing visits of physical therapy for supervision of exercises that are performed independently at home versus a single session of physical therapist instruction followed by an independent home program?
10. The use of a single injection of corticosteroids with local anesthetic can be considered for short-term improvement in both pain and function for patients with shoulder pain. In patients who cannot tolerate corticosteroids, injectable NSAIDs may be considered.	Is the use of a single injection of corticosteroids with local anesthetic considered for short-term improvement in both pain and function for patients with shoulder pain? Can injectable NSAIDs be considered in patients who cannot tolerate corticosteroids?
11. Prolotherapy is not recommended for use in patients with full-thickness rotator cuff tear.	Is prolotherapy recommended for use in patients with full-thickness rotator cuff tear?
12. Conversion to full-thickness or transtendinous/in-situ repair can be performed in patients that failed conservative management with high-grade partial-thickness rotator cuff tears.	Can patients with high-grade partial-thickness rotator cuff tears who failed conservative management be treated wth full-thickness or transtendinous/in-situ repair?
13. Debridement or repair of high-grade partial-thickness cuff tears that have failed physical therapy can be performed; however, repair of high-grade partial tears can improve outcomes.	Should debridement or repair of high-grade partial-thickness cuff tears be performed in patients that have failed physical therapy? Does repair of high-grade partial tears can improve outcomes?
14. Biological augmentation of rotator cuff repair with platelet-derived products is not recommended for improving patient-reported outcomes; however, limited evidence supports the use of liquid platelet rich plasma (PRP) in the context of decreasing retear rates.	Does biological augmentation of rotator cuff repair with platelet-derived products improve patient-reported outcomes? Is there evidence to support the use of liquid PRP in the context of decreasing retear rates?
15. Double row rotator cuff repair constructs are not recommended for improving patient-reported outcomes compared to single row repair constructs.	Are double row rotator cuff repair constructs recommended for improving patient-reported outcomes compared to single row repair constructs?
16. Double row repairs can result in lower overall retear rates after primary repair and improved patient-reported outcomes in large (>3cm) repairs. However, when evaluating for only full-thickness retears, double row repairs are not significantly favored.	Is there evidence in medical literature that double row repairs result in improved patient-reported outcomes and lower overall retear rates after primary repair in patients with large (>3cm) repairs? Is there evidence for this in full-thickness tears?
17. Marrow stimulation at the time of rotator cuff repair does not improve patient reported outcomes; however, this technique may decrease retear rates in patients with larger tear sizes.	Does marrow stimulation at the time of rotator cuff repair improve patient reported outcomes? Does this technique decrease retear rates in patients with larger tear sizes?
18. The use of human dermal allografts to augment rotator cuff repair can lead to lower retear rates and better patient-reported outcomes, but porcine allograft is not suggested for use in rotator cuff augmentation.	Does the use of human dermal allografts to augment rotator cuff repair lead to lower retear rates and better patient-reported outcomes? Is porcine allograft suggested for use in rotator cuff augmentation?
19. The use of bioinductive tendon implants to augment rotator cuff repair or as an alternative to non-augmented repair can lead to lower retear rates and better patientreported outcomes.	Can the use of bioinductive tendon implants to augment rotator cuff repair or as an alternative to non-augmented repair lead to lower retear rates and better patient reported outcomes?
20. Evidence shows no difference in long-term (> 1 year) patient-reported outcomes or cuff healing rates between open and arthroscopic repairs; however, arthroscopic-only technique is associated with better short-term improvement in post operative recovery of motion and decreased visual analog scale (VAS) scores.	Does the current evidence show a difference in long-term (> 1 year) patient-reported outcomes or cuff healing rates between open and arthroscopic repairs? Is arthroscopic-only technique associated with better short-term improvement in post operative recovery of motion and decreased visual analog scale (VAS) scores?
21. Multimodal analgesia programs or non-opioid individual modalities can be considered to provide added benefit for postoperative pain management following rotator cuff repair.	Can multimodal analgesia programs or non-opioid individual modalities be considered to provide added benefit for postoperative pain management following rotator cuff repair?
22. The use of hyaluronic acid injections may be considered in the non-operative management of rotator cuff pathology with no tears.	Can the use of hyaluronic acid injections be considered in the non-operative management of rotator cuff pathology with no tears?
23. The routine use of platelet rich plasma is not supported for the treatment of rotator cuff tendinopathy or partial tears.	Is the routine use of platelet rich plasma supported for the treatment of rotator cuff tendinopathy or partial tears?
24. Multiple steroid injections may compromise the integrity of the rotator cuff, which may affect attempts at subsequent repair.	Do multiple steroid injections compromise the integrity of the rotator cuff thus affecting attempts at subsequent repair?
25. Evidence suggests that the routine use of PRP in the non-operative management of fullthickness rotator cuff tears may not be indicated.	Is the routine use of PRP indicated in the non-operative management of full thickness rotator cuff tears?
26. In the absence of reliable evidence, it is the opinion of the workgroup that physical therapy, biceps tenotomy/tenodesis, partial repair, tendon transfer, superior capsular reconstruction, arthroscopic debridement, balloon spacers, graft interposition, or graft augmentation (non-porcine) can improve patient-reported outcomes.	Do physical therapy, biceps tenotomy/tenodesis, partial repair, tendon transfer, superior capsular reconstruction, arthroscopic debridement, balloon spacers, graft interposition, or graft augmentation (non-porcine) improve patient-reported outcomes?
27. In the absence of reliable evidence, it is the opinion of the workgroup that in patients with massive, unrepairable tears and significant functional loss who have failed other treatments, reverse arthroplasty can improve patient-reported outcomes.	Can reverse arthroplasty improve patient-reported outcomes in patients with massive, unrepairable tears and significant functional loss who have failed other treatments?
28. In the absence of reliable evidence, it is the opinion of the workgroup that after failure of conservative treatment, reverse shoulder arthroplasty for massive unrepairable tears with glenohumeral joint arthritis can improve patient-reported outcomes.	Can reverse shoulder arthroplasty for massive unrepairable tears with glenohumeral joint arthritis improve patient-reported outcomes in patients that failed conservative treatment?
29. In the absence of reliable evidence, it is the opinion of the workgroup that physical therapy can improve outcomes in patients with low-grade or intermediate-grade partial-thickness rotator cuff tears. In patients with persistent pain and functional impairment after appropriate non-operative treatment, surgery can improve outcomes.	Can physical therapy improve outcomes in patients with low-grade or intermediate-grade partial-thickness rotator cuff tears? In patients with persistent pain and functional impairment after appropriate non-operative treatment, can surgery improve outcomes?
Developmental Dysplasia of the Hip	
1. Moderate evidence supports not performing universal ultrasound screening of newborn infants.	Does the current medical literature support performing universal ultrasound screening of newborn infants?
2. Strong evidence supports performing an imaging study before 6 months of age in infants with one or more of the following risk factors: breech presentation, family history, or history of clinical instability.	Is there evidence to support performing an imaging study before 6 months of age in infants with one or more of the following risk factors: breech presentation, family history, or history of clinical instability?
3. Limited evidence supports that the practitioner might obtain an ultrasound in infants less than 6 weeks of age with a positive instability examination to guide the decision to initiate brace treatment.	Does the current literature support practitioners obtaining an ultrasound in infants less than 6 weeks of age with a positive instability examination to guide the decision to initiate brace treatment?
4. Limited evidence supports the use of an AP pelvis radiograph instead of an ultrasound to assess developmental dysplasia of the hip in infants beginning at 4 months of age.	Should AP pelvis radiograph or an ultrasound be used to assess developmental dysplasia of the hip in infants beginning at 4 months of age?
5. Moderate evidence supports that a practitioner re-examine infants previously screened as having a normal hip examination on subsequent visits prior to 6 months of age.	Should practitioners re-examine infants previously screened as having a normal hip examination on subsequent visits prior to 6 months of age?
6. Limited evidence supports observation without a brace for infants with a clinically stable hip with morphologic ultrasound imaging abnormalities.	Is observation without a brace sufficient for infants with a clinically stable hip despite morphologic ultrasound imaging abnormalities?
7. Limited evidence supports either immediate or delayed (2-9 weeks) brace treatment for hips with a positive instability exam.	In patients with a positive instability exam, is there evidence to support immediate or delayed (2-9 weeks) brace treatment?
8. Moderate evidence supports use of the von Rosen splint over Pavlik, Craig, or Frejka splints for initial treatment of an unstable hip.	Which splint is preferred for initial treatment of infants with an unstable hip: the von Rosen splint, Pavlik splint, Craig splint, or Frejka splint.
9. Limited evidence supports that the practitioner perform serial physical examinations and periodic imaging assessments (ultrasound or radiograph based on age) during management for unstable infant hips.	Should serial physical examinations and periodic imaging assessments (ultrasound or radiograph based on age) be performed during management for unstable infant hips?
Orthopedic Oncology	
1. In the absence of reliable evidence, it is the opinion of the workgroup that the combination of imaging findings and lesion-related pain is predictive of risk of pathologic femur fracture. There is no reliable evidence to suggest that MRI is a strong predictor of femur fracture.	Is the combination of imaging findings and lesion-related pain predictive of risk of pathologic femur fracture? Is there reliable evidence to suggest that MRI is a strong predictor of femur fracture?
2. In the absence of reliable evidence, it is the opinion of the workgroup that the use of bone modifying agents may assist in reducing incidence of femur fractures in patients with metastatic carcinoma or multiple myeloma and bone lesions.	Does the use of bone modifying agents assist in reducing incidence of femur fractures in patients with metastatic carcinoma or multiple myeloma and bone lesions?
3. Clinicians should consider decreasing the frequency of zoledronic acid dosing to 12 weeks (compared to the standard 4-week interval), as this is associated with non-inferior SRE outcomes and similar adverse event rates in patients with metastatic carcinoma or multiple myeloma. Clinicians should consider long-term use of bone modifying agents to reduce skeletal related events in patients with multiple myeloma.	Is reduction of the frequency of zoledronic acid dosing to 12 weeks (compared to the standard 4-week interval) associated with non-inferior SRE outcomes and similar adverse event rates in patients with metastatic carcinoma or multiple myeloma? Should clinicians consider long-term use of bone modifying agents to reduce skeletal related events in patients with multiple myeloma?
4. In the absence of reliable evidence, it is the opinion of the workgroup that bone modifying agents should be considered in patients with metastatic carcinoma or multiple myeloma with bone lesions at risk for fracture regardless of tumor histology.	Should bone modifying agents be considered in patients with metastatic carcinoma or multiple myeloma with bone lesions at risk for fracture regardless of tumor histology?
5. In the absence of reliable evidence, it is the opinion of the workgroup that imaging findings of lateral cortical thickening may be associated with increased atypical femur fracture risk.	Are imaging findings of lateral cortical thickening associated with increased atypical femur fracture risk?
6. Clinicians should consider the use of radiation therapy to decrease the rate of femur fractures in patients with metastatic carcinoma or multiple myeloma lesions who are deemed at increased risk based on the combination of imaging findings and lesion-related pain.	Does the use of radiation therapy decrease the rate of femur fracture in patients with metastatic carcinoma or multiple myeloma at increased risk for fracture?
7. In the absence of reliable evidence, it is the opinion of the workgroup that clinicians may consider the use of radiation therapy in patients undergoing prophylactic femur stabilization to reduce pain, improve functional status, and reduce the need for further intervention.	Based on the current medical literature, should clinicians consider the use of radiation therapy in patients undergoing prophylactic femur stabilization to reduce pain, improve functional status, and reduce the need for further intervention?
8. In the absence of reliable evidence, it is the opinion of the workgroup that radiation therapy may be considered after resection and reconstruction to reduce pain, improve functional status, and reduce the need for further intervention in patients with residual tumor, or those at increased risk of tumor recurrence.	Should radiation therapy be considered after resection and reconstruction to reduce pain, improve functional status, and reduce the need for further intervention in patients with residual tumor, or those at increased risk of tumor recurrence?
9. Clinicians should consider the use of multi-fraction in lieu of single fraction radiation treatment to reduce the risk of fracture in patients with metastatic carcinoma in the femur.	Is multi-fraction radiation better than single fraction radiation for reducing fracture risk in patients with metastatic carcinoma of the femur?
10. In the absence of reliable evidence, it is the opinion of the workgroup that surgeons utilize a validated method of estimating survival of the patient in choosing the method of reconstruction. Longer survival estimates may justify more durable reconstruction methods such as arthroplasty, if clinically appropriate.	Based on the current evidence, should surgeons utilize a validated method of estimating survival of the patient in choosing the method of reconstruction. Do longer survival estimates justify more durable reconstruction methods such as arthroplasty?
11. In the absence of reliable evidence, it is the opinion of the workgroup that when treating a femoral neck fracture with hemiarthroplasty, use of a long stem can be associated with increased intra-operative and post-operative complications and should only be used in patients with additional lesions in the femur.	When treating a femoral neck fracture with hemiarthroplasty, is a long stem be associated with increased intra-operative and post-operative complications? Should a long stem be used to treat a femoral neck fracture with hemiarthroplasty in patients with additional lesions in the femur?
12. In the absence of reliable evidence, it is the opinion of the workgroup that there is no advantage to routine use of cephalomedullary nails for diaphyseal metastatic lesions as there does not appear to be a high frequency of new femoral neck lesions following intramedullary nailing.	Is there an advantage to routine use of cephalomedullary nails for diaphyseal metastatic lesions, even if there does not appear to be a high frequency of new femoral neck lesions following intramedullary nailing?
13. Clinicians may consider arthroplasty to improve patient function and decrease the need for post-operative radiation therapy in patients with pathologic fractures from metastatic carcinoma in the femur.	Does arthroplasty improve patient function and decrease the need for post-operative therapy in patients with pathologic fractures from metastatic carcinoma in the femur?

Appendix C 

**Table 4:** Total count of incorrects per citation element separated by the four Clinical Practice Guidelines (CPGs) analyzed. DDH: developmental dysplasia of the hip

Citation metric	Hip fracture (n = 95)	Mean/query	Rotator cuff (n = 145)	Mean/query	DDH	Mean/query	Oncology (n = 65)	Mean/query	Total (n = 350)	Mean/query
Total hallucinated/fabricated	33 (4.58%)	0.35	125 (10.04%)	0.86	7 (2.46%)	0.16	30 (6.17%)	0.46	195 (7.13%)	0.56
Total references	721	7.59	1245	8.59	284	6.31	486	7.48	2736	7.82
Total title incorrect	216 (29.96%)	2.27	390 (31.33%)	2.69	80 (28.17%)	1.78	154 (31.69%)	2.37	840 (30.70%)	2.4
Total author incorrect	122 (16.92%)	1.28	205 (16.47%)	1.41	46 (16.20%)	1.02	71 (14.61%)	1.09	444 (16.23%)	1.27
Total journal incorrect	110 (15.26%)	1.16	278 (22.33%)	1.92	47 (16.55%)	1.04	82 (16.87%)	1.26	517 (18.90%)	1.48
Total year incorrect	28 (3.88%)	0.29	120 (9.64%)	0.83	20 (7.04%)	0.44	30 (6.17%)	0.46	198 (7.24%)	0.57
Total pages incorrect	165 (22.88%)	1.74	380 (30.52%)	2.62	71 (25.00%)	1.58	136 (27.98%)	2.09	752 (27.49%)	2.15
Total volume incorrect	152 (21.08%)	1.6	357 (28.67%)	2.46	66 (23.24%)	1.47	125 (25.72%)	1.92	700 (25.58%)	2
Total PMID incorrect	192 (26.63%)	2.02	458 (36.79%)	3.16	139 (48.94%)	3.09	231 (47.53%)	3.55	1020 (37.28%)	2.91
Total correct citations	399 (55.34%)	4.2	618 (49.64%)	4.26	122 (42.96%)	2.71	211 (43.42%)	3.25	1350 (49.34%)	3.86
Queries in which all citations are correct	10 (10.53%)	0.11	14 (9.66%)	0.1	0 (0.00%)	0	4 (6.15%)	0.06	28 (8.00%)	0.08
